# Ultrahigh Piezoelectric Performance through Synergistic Compositional and Microstructural Engineering

**DOI:** 10.1002/advs.202105715

**Published:** 2022-03-16

**Authors:** Yongke Yan, Liwei D. Geng, Li‐Feng Zhu, Haoyang Leng, Xiaotian Li, Hairui Liu, Dabin Lin, Ke Wang, Yu U. Wang, Shashank Priya

**Affiliations:** ^1^ Department of Materials Science and Engineering Pennsylvania State University University Park PA 16802 USA; ^2^ Department of Materials Science and Engineering Michigan Technological University Houghton MI 49931 USA; ^3^ Materials Research Institute Pennsylvania State University University Park PA 16802 USA

**Keywords:** local structural heterogeneity, phase‐field simulations, piezoelectric ceramics, texturing

## Abstract

Piezoelectric materials enable the conversion of mechanical energy into electrical energy and vice‐versa. Ultrahigh piezoelectricity has been only observed in single crystals. Realization of piezoelectric ceramics with longitudinal piezoelectric constant (*d*_33_) close to 2000 pC N^–1^, which combines single crystal‐like high properties and ceramic‐like cost effectiveness, large‐scale manufacturing, and machinability will be a milestone in advancement of piezoelectric ceramic materials. Here, guided by phenomenological models and phase‐field simulations that provide conditions for flattening the energy landscape of polarization, a synergistic design strategy is demonstrated that exploits compositionally driven local structural heterogeneity and microstructural grain orientation/texturing to provide record piezoelectricity in ceramics. This strategy is demonstrated on [001]_PC_‐textured and Eu^3+^‐doped Pb(Mg_1/3_Nb_2/3_)O_3_‐PbTiO_3_ (PMN‐PT) ceramics that exhibit the highest piezoelectric coefficient (small‐signal *d*_33_ of up to 1950 pC N^–1^ and large‐signal *d*_33_* of ≈2100 pm V^–1^) among all the reported piezoelectric ceramics. Extensive characterization conducted using high‐resolution microscopy and diffraction techniques in conjunction with the computational models reveals the underlying mechanisms governing the piezoelectric performance. Further, the impact of losses on the electromechanical coupling is identified, which plays major role in suppressing the percentage of piezoelectricity enhancement, and the fundamental understanding of loss in this study sheds light on further enhancement of piezoelectricity. These results on cost‐effective and record performance piezoelectric ceramics will launch a new generation of piezoelectric applications.

## Graphical abstract

Guided by theoretical models and phase‐field simulations that provide conditions for flattening the energy landscape of polarization, a synergistic design strategy that exploits compositionally driven local structural heterogeneity and grain orientation/texturing is demonstrated to provide record piezoelectric coefficients of 2000 pC N^–1^ in piezoelectric ceramics. These results will have significant impact on the current and future piezoelectric applications.

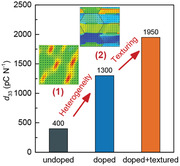

## 1 Introduction

Piezoelectric ceramic materials with high piezoelectric constant can generate large strain in response to applied electric field or generate high voltage in response to applied mechanical stress. Piezoelectric ceramics find application in variety of electronic, communication, imaging, sensing and electromechanical systems.^[^ ^1^, ^2^, ^3^ ^]^ High energy density and ease of miniaturization are further driving the development of new piezoelectric technologies, such as energy harvesters for the Internet of Things (IoTs) devices.^[^ ^3^ ^]^ Among all the known piezoelectric materials, perovskite ferroelectrics exhibit the highest longitudinal piezoelectric strain/charge coefficient, *d*_33_. In perovskite ferroelectrics, the longitudinal piezoelectric coefficient *d*_33_ can be expressed as *d*_33_ =  2*Q*_33_ *P*_r_ *ε*_33_, where *Q*_33_ is the electrostrictive coefficient, *P*_r_ is the remanent polarization, and *ε*_33_is the dielectric permittivity.^[^ ^4^, ^5^ ^]^ The most widely adopted approach for enhancing the piezoelectric response *d*_33_ is by increasing the dielectric permittivity *ε*_33_ via flattening of the Gibbs free energy landscape with respect to the polarization (lowering the energy barrier for ferroelectric polarization rotation). The traditional method to flatten energy landscape is by designing composition‐induced multiphase region, such as morphotropic phase boundary (MPB) or polymorphic phase transition (PPT).^[^ ^6^, ^7^ ^]^ There are two emerging approaches to further improve the piezoelectric response, namely, compositional local structural heterogeneity and microstructural texture engineering.

The approach of compositional local structural heterogeneity engineering is to create heterogenous polar nanoregions by means of either nanoscale B‐site cation ordering (for example, Mg^2+^ and Nb^5+^ short range ordering in PMN) or A‐site aliovalent doping (such as, Sm^3+^ doped PMN‐PT, and La^3+^ doped Pb(Zr,Ti)O_3_, PLZT), and hence introduce interfacial energies associated with local polarization and strain discontinuities.^[^ ^5^, ^8^, ^9^, ^10^, ^11^, ^12^, ^13^ ^]^ The competition between the matrix energy and the interfacial energies can flatten the energy landscape to increase the dielectric permittivity and piezoelectricity.^[^ ^12^ ^]^ The approach of local structural heterogeneity engineering has been demonstrated in recent breakthrough in Sm^3+^‐doped Pb(Mg_1/3_Nb_2/3_)O_3_‐PbTiO_3_ (PMN‐PT) materials which exhibit ultrahigh longitudinal piezoelectric coefficients *d*_33_ of ≈1500 pC N^–1^ in ceramics.^[^ ^12^ ^]^ The introduction of Sm^3+^ dopant on the A‐site of PMN‐PT lattice created local structural heterogeneity and disruptions in the long‐range ferroelectric domains, resulting in significant increase in both dielectric permittivity and piezoelectric response.^[^ ^12^, ^13^ ^]^

The approach of microstructural texture engineering in ceramics is to utilize the strong intrinsic anisotropy of piezoelectric properties available in single crystals. For example, the *d*_33_ of up to 2820 pC N^–1^ in [001]_PC_‐oriented rhombohedral PMN‐PT single crystals is nearly 15 times larger than the *d*_33_‐value (190 pC N^–1^) along <111>_PC_ direction.^[^ ^14^ ^]^ Since the practical application of single crystals is challenged by their relatively higher cost, time‐consuming synthesis process, and composition inhomogeneity due to incongruent melting issues, the most widely used piezoelectric materials are based on polycrystalline ceramics with random grain orientations exhibiting average piezoelectric properties. Considering the beneficial characteristic of anisotropy in polycrystalline materials, extensive efforts have been made toward developing crystallographic textured polycrystalline ceramics, referring to ceramics with large degree of grains oriented along a preferred crystallographic direction. A wide variety of textured piezoelectric ceramics have been reported with enhanced piezoelectric properties ^[^ ^7^, ^15^, ^16^, ^17^, ^18^, ^19^, ^20^, ^21^, ^22^ ^]^. For example, the longitudinal piezoelectric coefficient, *d*_33_, of [001]_PC_ textured PMN‐PT and PMN‐PZT ceramics was found to exceed 1000 pC N^–1^, which is about two to five times higher than that of the random ceramics.^[^ ^15^, ^16^ ^]^

The aforementioned compositional local structural heterogeneity and microstructural texture engineering have been individually studied and much progress has been made based on these strategies. However, it has been challenging to implement the synergistic design that exploits both these mechanism in the same material. One of the main challenges in achieving this synergistic approach is that microstructural design is significantly influenced by composition induced local structural heterogeneity. This mutual coupling presents resistance in obtaining the desired local structure and microstructure. Here, we demonstrate for the first‐time success in resolving this challenge which resulted in record piezoelectric performance in ceramics.

## 2 Results and Discussion

### 2.1 Synergistic Design Strategy for Ultrahigh Piezoelectricity in Ceramics

**Figure 1:**
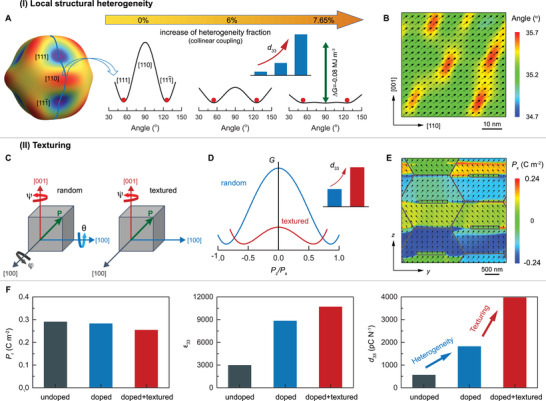
Phenomenological illustration and phase‐field simulation of enhanced piezoelectricity via local structural heterogeneity and texture engineering. A) Landau free energy of ferroelectrics with different volume fraction of the local structural heterogeneity. B) Phase‐field simulated polarization distribution of PMN‐PT with 5% heterogeneities. C) Schematic diagram of grain orientation distributions in random and textured polycrystals. D) Landau free energy profiles with respect to the polarization component *P_z_* (normalized by saturated polarization *P*_s_) along poling direction *z* for random and textured polycrystals, where the inset shows the corresponding *d*_33_ increment. E) Phase‐field simulated polarization distribution of [001]_PC_‐textured PMN‐PT polycrystal with 5% heterogeneities (doped) after electrical poling along *z*‐direction. F) Phase‐field simulation of the remanent polarization *P*_r_, dielectric permittivity *ε*_33_, and longitudinal piezoelectric coefficient *d*_33_ for undoped, doped, and doped+textured polycrystals. Note: *x*, *y*, and *z* are defined in the “lab coordinate” while directions labeled with square brackets are defined in the “crystallographic coordinate,” and *z* is the poling direction that coincides with [001] for [001]_PC_‐textured polycrystal in our simulations.

Guided by phenomenological models and phase‐field simulations, we propose a synergistic approach of local structural heterogeneity and grain texturing to enhance piezoelectricity via flattening the energy landscape of polarization. Local structural heterogeneity was introduced by doping rare‐earth elements in classical relaxor ferroelectrics, and its fraction depends on the concentration of dopants. It is complex to exactly quantify the percentage of local structural heterogeneity just by taking into account the doping amount of rare‐earth elements. Rather a good understanding of the underlying physical mechanisms is needed. Therefore, to explore the doping effect on piezoelectricity, we invoked Landau theory to examine how the piezoelectric coefficient *d*_33_ changes with the volume fraction of local heterogeneities. **Figure** **1**A illustrates the enhancement of *d*_33_ via the increment of heterogeneity volume fraction for PMN‐PT. In this work, PMN‐PT is in rhombohedral phase with the spontaneous polarization along <111> directions at room temperature. The inclusion of heterogeneity that favors the orthorhombic phase (polarization along <110> directions) can flatten the energy landscape because of the competition between bulk and interfacial energy. According to the phase‐field simulation in Figure 1B, the coupling between matrix and heterogeneities is collinear at room temperature,^[^ ^23^ ^]^ where the polarization in heterogeneities is almost parallel with that in matrix to reduce the interfacial energies. Such a collinear behavior can be examined from the angle between polarization and [110]‐direction. These “collinear” heterogeneities will facilitate the polarization rotation of nearby regions and result in a highly fattened energy profile, leading to increased permittivity and piezoelectricity. The detailed phase‐field simulation results on the effect of heterogeneity fraction on *d*_33_ are shown in Figure S1 (Supporting Information). The simulations reveal that the elevated fraction of heterogeneities can effectively increase *d*_33_ until reaching a critical point beyond which the heterogeneity's become dominant and *d*_33_ begins to drop.

Texturing can further increase the piezoelectricity of rare‐earth doped PMN‐PT, as illustrated in Figure 1C,D. The texturing technique enables grains to align with each other along a specific crystallographic orientation, which enables design of polycrystalline ceramics that outperform traditional ceramics by exhibiting piezoelectric performance comparable or even equal to that of single crystals. Figure 1C schematically illustrates the grain orientation distributions in random and textured polycrystals. For the random polycrystal, all the grain orientations are randomly distributed. For the [001]_PC_‐textured polycrystal, the grains are oriented along the [001]_PC_ crystallographic axis with [010]_PC_ and [100]_PC_ crystallographic axes distributed randomly. For both random and textured polycrystals, once they are poled along a certain direction (*z*‐direction), the eight <111> directions become nonequivalent and the spontaneous polarizations in each grain prefers to stay as close to the poling direction as possible. Herein, *x*, *y*, and *z* are defined in the “lab coordinate” while directions labeled with square brackets are defined in the “crystallographic coordinate,” and *z* is the poling direction that coincides with [001] for [001]_PC_‐textured polycrystal in our simulations. If neglecting the correlation between grains, the average polarization in the poling direction is $P_{z}/P_{s}=\sqrt{3}\;/2$ for random polycrystal and $P_{z}/P_{s}=\;1/\sqrt{3}$ for [001]‐textured polycrystal, as indicated by the energy‐minimum points in Figure 1D. It is worth noting that the average angle between polarization and poling direction is 30° for random polycrystal and 54.74° for textured (which is the maximum allowed angle for poled rhombohedral ferroelectrics). Since the piezoelectric response for relaxor‐PT crystals is related to “polarization rotation” rather than “polarization elongation,”^[^ ^24^ ^]^ textured polycrystal with the larger angle will exhibit a higher permittivity and piezoelectricity as compared to random polycrystal. According to the equivalent energy profiles in Figure 1D, the latter is found to have a flatter energy landscape and thus a higher *d*_33_.

To examine the synergistic design of local structural heterogeneity and texturing, we performed phase‐field simulations for undoped (random polycrystal), doped (random polycrystal with heterogeneity), and doped + textured (textured polycrystal with heterogeneity) PMN‐PT ceramics. As discussed above, under a poling treatment, local polarizations will be aligned along the spontaneous polarization directions that are closer to the poling direction. Figure 1E shows the simulated polarization distribution of [001]_PC_‐textured PMN‐PT polycrystal with 5% heterogeneities (doped) after electrical poling along *z*‐direction. Upon poling, there are four equivalent polarization directions for the rhombohedral phase, which form a “stripe” domain structure, in analogy to stripe domains separated by 109° domain walls in single crystals. This single‐crystal domain feature present in polycrystals results from the fixed [001] crystallographic axis. However, since the other axes are free and randomly distributed, the electrical and elastic correlations between neighboring grains still exist, which makes the polarizations deviate from their original directions and thus results in imperfect “stripe” domains with non‐109° domain walls. Please note that such a stripe‐like domain structure will not be present in random polycrystals (Figure S2, Supporting Information). The nucleation of these “stripe” domain walls usually takes place from grain boundaries as well as the tetragonal‐phase BaTiO_3_ templates. Figure 1F shows the simulated polarization remanence *P*_r_, dielectric permittivity *ε*_33_, and piezoelectric coefficient *d*_33_ for the three types of ceramics, undoped (random polycrystal), doped (random polycrystal with heterogeneity), and doped + textured (textured polycrystal with heterogeneity). Piezoelectric coefficient *d*_33_ is found to be significantly increased through the local structural heterogeneity and texture engineering. For heterogeneity engineering, the enhancement of *d*_33_ is mainly attributed to the increment of *ε*_33_. For texture engineering, *ε*_33_ is increased, resulting in the enhancement of *d*_33_. These findings suggest that ultrahigh piezoelectricity can be achieved in polycrystal ceramics by following the proposed synergistic design.

### 2.2 Multiscale Microstructures of Synthesized [001]_PC_‐Textured and Eu^3+^‐Doped PMN–PT Ceramics

**Figure 2:**
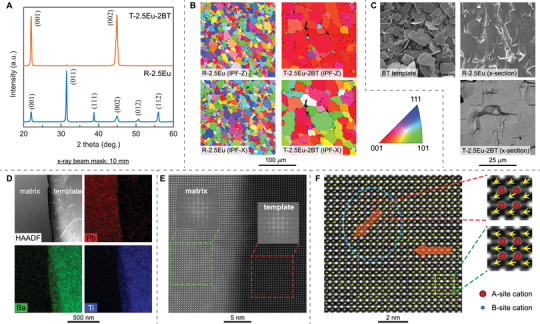
Multiscale microstructures of random and textured Eu^3+^‐doped PMN‐PT at different scales (cm to nm). A) X‐ray diffraction patterns of random and textured Eu^3+^‐doped PMN‐PT ceramics, abbreviated as R‐2.5Eu and T‐2.5Eu‐2BT, respectively. B) Inverse pole Figure (IPF) maps of R‐2.5Eu and T‐2.5Eu‐2BT ceramics, measured by the SEM‐EBSD technique. The IPF components in Z and X directions reveal the sample's out‐of‐plane (normal) and the in‐plane spatial grain orientation distributions. C) SEM images of [001] BaTiO_3_ templates and cross‐sections (x‐section) of R‐2.5Eu and T‐2.5Eu‐2BT ceramics. D) Element distribution mappings of T‐2.5Eu‐2BT textured ceramics, measured by STEM‐EDS technique. E) High resolution STEM image of template/matrix interface in T‐2.5Eu‐2BT textured ceramics. F) HR‐STEM images of T‐2.5Eu‐2BT matrix recorded along the crystallographic [001]_PC_ direction. The polar vectors (arrows) are given for each unit‐cell column in the atomic resolution STEM. The positions of the A‐site and B‐site atomic columns are indicated in the enlarged images on the right.

To experimentally implement the proposed synergistic design, PMN‐PT system is selected, as significant improvement of piezoelectricity has been demonstrated individually in rare‐earth doped PMN‐PT system ^[^ ^25^ ^]^ and textured PMN‐PT ceramics.^[^ ^15^ ^]^ For a better understanding of dielectric, ferroelectric and piezoelectric behavior of PMN‐PT system with synergistic strategy of the heterogeneity and texturing techniques, highly [001]_PC_‐textured Eu^3+^‐doped PMN‐PT piezoelectric ceramics were prepared by templated grain growth technique using [001]_PC_ BaTiO_3_ templates (Eu^3+^ was added in the form of Eu_2_O_3_, please refer to Materials and Methods section for synthesis details). **Figure** **2** depicts the microstructures of 2.5 mol% Eu^3+^‐doped and [001]_PC_‐textured PMN‐28PT ceramics with 2 vol% BaTiO_3_ templates on a variety of length scale from cm to nm, abbreviated as T‐2.5Eu‐2BT. For comparison, the random counterparts were prepared by the same processing method but without adding templates, abbreviated as R‐2.5Eu. Figure 2A shows the X‐ray diffraction patterns for T‐2.5Eu‐2BT and R‐2.5Eu ceramics, respectively. Both samples exhibit perovskite phase, while textured ceramics show a remarkable enhancement in the intensity of the (001) diffraction peak compared to random ceramics. The Lotgering factor of the textured sample is over 98%, indicating a strong [001]_PC_ preferred grain orientation. Electron backscatter diffraction (EBSD) was performed to further identify the crystallographic orientation of each grain in the textured sample. From Figure 2B, it can be observed that the grain orientation in random sample are randomly distributed in three‐dimensions, while the [001]_PC_‐oriented grains in textured sample are well aligned along the thickness direction (*z*, out of casting plane). Further, the [100]_PC_ and [010]_PC_ orientations of grains in textured samples are randomly distributed in the casting plane. This is related to the fact that unidirectional shear force was used for aligning the templates. As shown in Figure 2C, BaTiO_3_ template shows a plate‐like morphology, with length of 10–20 µm and thickness of 0.5–1 µm, respectively. These high‐aspect‐ratio BaTiO_3_ platelets can be well aligned in the matrix during the tape casting via the shear force applied by doctor blade. The 2.5Eu‐doped PMN‐28PT matrix epitaxially grew from these aligned BaTiO_3_ templates (Figure 2C), yielding strong [001] orientation out of the casting plane. Interfacial regions among BaTiO_3_ template and 2.5Eu‐PMN‐28PT matrix were investigated by high resolution scanning transmission electron microscope (HR‐STEM) and energy dispersive spectrometry (EDS) element mapping, as shown in Figure 2D,E. The sharp interface indicates a negligible inter‐diffusion between template and matrix. The atomic arrangement, characterized by fast Fourier transform (FFT) diffraction patterns from HR‐STEM, indicates the epitaxial growth along [001]_PC_ orientation, which is the origin of textured structure. With the similar approach for identifying the local structural heterogeneity in Sm‐doped PMN‐PT,^[^ ^12^ ^]^ the positions of the A‐site and B‐site atomic columns in [001]_PC_‐textured and Eu^3+^‐doped PMN‐PT ceramics are depicted by HR‐STEM as shown in Figure 2E. According to the atomic positions in this Figure 2F, the atomic displacements are presented as vectors pointing from the center of a B‐site cation to the center of its four nearest neighboring A‐site cations. These atomic displacements represent the magnitudes and directions of the polar vectors for each unit‐cell column. The HR‐STEM images clearly reveal the existence of local structural heterogeneity at the nanoscale in [001]_PC_‐textured and Eu^3+^‐doped PMN‐PT ceramics.

### 2.3 Piezoelectricity of [001]_PC_‐Textured and Eu^3+^‐Doped PMN–PT Ceramics

**Figure 3:**
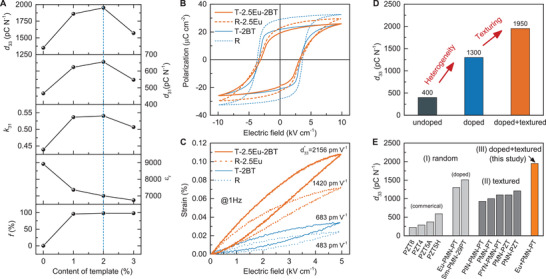
Piezoelectric and dielectric properties of [001]_PC_‐textured and Eu^3+^‐doped PMN‐PT ceramics. A) Texture degree *f*, dielectric constant *ε*_r_, electromechanical coupling *k*_31_, piezoelectric coefficient *d*_31_ and *d*_33_ of 2.5 mol% Eu^3+^‐doped PMN‐PT with different contents of BaTiO_3_ templates. B) Polarization‐electric field loops of random PMN‐PT (R), PMN‐PT textured ceramics with 2 vol% BT template (T‐2BT), random Eu^3+^‐doped PMN‐PT (R‐2.5Eu), and 2.5 mol% Eu‐doped PMN‐PT textured ceramics with 2 vol% BT template (T‐2.5Eu‐2BT), measured at 1 Hz. C) Electric‐field‐induced strain of R, T, R‐2.5, and T‐2.5Eu‐2BT ceramics, measured at 1 Hz. D) Comparison of piezoelectric coefficient *d*_33_ between undoped (R), doped (R‐2.5Eu) and doped+textured (T‐2.5Eu‐2BT) ceramics. E) Comparison of piezoelectric coefficient *d*_33_ between commercial PZT ceramics, reported lead‐based textured ceramics and Eu^3+^‐doped PMN‐PT textured ceramics (T‐2.5Eu‐2BT). References are listed in the Supporting Information.

**Figure** **3**A shows the dielectric and piezoelectric properties of [001]_PC_‐textured 2.5Eu‐PMN‐PT ceramics with different amounts of BaTiO_3_ templates. It can be observed that texturing degree *f* reaches 96% even with 1 vol% templates and slightly increases with further increasing template content. In contrast, the dielectric constant *ε*_r_ decreases with template content due to the low permittivity of BaTiO_3_ (*ε*_*r*_ = 130) along [001]_PC_ direction ^[^ ^26^ ^]^). This phenomenon has also been observed in textured PMN‐PT without Eu^3+^ doping.^[^ ^15^ ^]^ Piezoelectric coefficient *d*_33_ shows a peak value of 1950 pC N^–1^ with 2 vol% template, which is different from the monotonous change of texture degree *f* and dielectric constant *ε*_r_. To understand this behavior, the equation for *d*_33_ =  2*Q*_33_ *P*_r_ *ε*_33_ is discussed here again. The piezoelectric coefficient *d*_33_ is strongly related to the variation of *Q*_33_, *P*_r_ and *ε*_33_. The electrostrictive coefficient *Q*_33_ of perovskite ferroelectrics is highly anisotropic. The maximum electrostrictive coefficient *Q*_33_ is along the <001>_PC_ directions, while the moderate and minimum values are along the <011>_PC_ and <111>_PC_ directions, respectively.^[^ ^27^ ^]^ It is therefore concluded that higher [001]_PC_ texturing degree will generate larger *Q*_33_. In regards to *P*_r_, theoretically, the intrinsic value for the rhombohedral phase follows the relationship *P*_r, [001]PC_ = *P*_r, <111>PC_ $/\sqrt{3}$. It can be expected that *P*_r_ decreases with the increase of [001]_PC_ texturing degree in textured ceramics. This trend has been observed in this study (Figure 3B) and previous studies.^[^ ^28^, ^29^, ^30^ ^]^ Therefore, the opposite trend of *ε*_r_ and *P*_r_, and *Q*_33_ change with increase in BaTiO_3_ template content, provides a peak point in piezoelectric properties with 2 vol% BT template. Figure 3C shows the electric‐field‐induced strain for undoped/doped and random/textured ceramics, where it can be observed that both the addition of Eu^3+^ dopant and [001]_PC_ texturing improves the strain and the corresponding large‐signal piezoelectric coefficient $d_{33}^{*}$. Figure 3D shows the changes in small‐signal *d*_33_ with Eu^3+^ doping and texturing, and the experimental result is consistent with the theoretical prediction as shown in Figure 1C. Through the combination of Eu^3+^ doping and texture engineering, the highest *d*_33_ of 1950 pC N^–1^ is achieved. Figure 3E compares the piezoelectric coefficient *d*_33_ among different types of piezoelectric ceramics, including PZT ceramics,^[^ ^31^ ^]^ reported lead‐based textured ceramics ^[^ ^15^, ^16^, ^22^, ^32^ ^]^ and Eu^3+^‐doped PMN‐PT textured ceramics (T‐2.5‐2BT) designed in this study. From this figure, it can be observed that the Eu^3+^‐doped PMN‐PT textured ceramics (T‐2.5‐2BT) synthesized in this study have the highest values among all the published piezoelectric ceramics.

### 2.4 Loss in [001]_PC_‐Textured and Eu^3+^‐Doped PMN–PT Ceramics

**Figure 4:**
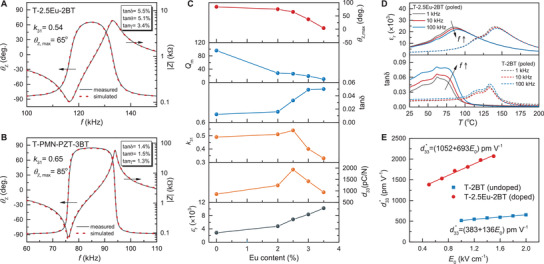
Losses of [001]_PC_‐textured and Eu^3+^‐doped PMN‐PT ceramics. Impedance |Z| and impedance phase angle *θ*_Z_ spectra of A) T‐2.5Eu‐2BT textured ceramics and B) T‐PMN‐PZT‐3BT textured ceramics. Here, the dielectric loss tan *δ*, piezoelectric loss tan *θ*, and electromechanical loss tan*γ* were fitted using loss incorporated equivalent circuit. C) Dielectric and piezoelectric properties of textured PMN‐PT ceramics with different contents of Eu^3+^ dopant. D) Temperature‐dependent dielectric constant *ε*_r_ and dielectric loss tan *δ* of textured T‐2BT and T‐2.5Eu‐2BT ceramics. E) Rayleigh behavior for *d*_33_* as a function of amplitude *E*_0_ at 1 Hz.

Although the piezoelectric coefficient *d*_33_ of [001]_PC_‐textured Eu^3+^‐doped PMN‐PT (T‐2.5Eu‐2BT) ceramics possess the highest value among all reported piezoelectric ceramics, the percentage of piezoelectricity enhancement from texturing is only 50% (from 1300 pC N^–1^ in R‐2.5Eu to 1950 pC N^–1^ in T‐2.5Eu‐2BT), which is smaller than the value expected based on the theoretical prediction (Figure 1C) and previous experimental results reported in other compositions. Experimentally, over 100% improvement has been widely achieved in a variety of compositions, such as PMN‐PT, PMN‐PZT,^[^ ^16^ ^]^ BNT‐BT,^[^ ^33^ ^]^ KNN,^[^ ^20^ ^]^ BCZT,^[^ ^21^ ^]^ and PIN‐PMN‐PT.^[^ ^22^ ^]^ Therefore, the origin of limited enhancement of piezoelectric coefficient in highly [001]_PC_‐textured and Eu^3+^‐doped PMN‐PT ceramics is necessary to be investigated. There is another observation that the coupling coefficient *k*_31_ of textured and Eu^3+^‐doped PMN‐PT ceramics is only 0.54, which is much lower than the values reported for textured PMN‐PT (0.60) and textured PMN‐PZT (0.65). According to the relation: $d_{31}=k_{31}\;\sqrt{s_{33}\varepsilon_{33}}$, high *k*_31_ will result in high *d*_31_. Since the texturing degree of textured and Eu^3+^‐doped PMN‐PT is over 96%, which is similar to the values for textured PMN‐PT and textured PMN‐PZT, the lower *k*_31_ should be related to composition instead of texturing degree. As indicated by impedance spectra in **Figure** **4**A,B, the relatively lower *k*_31_ in T‐2.5Eu‐2BT is related to the low impedance phase angle *θ*_Z,max_. Based upon the coupled electrical‐mechanical constitutive relations at resonance, it is well‐known that the impedance phase angle *θ*_Z,max_ is usually related to low poling degree, induced by various losses, including dielectric loss tan *δ*, piezoelectric loss tan*θ*, and electromechanical loss tan*γ*.^[^ ^34^, ^35^, ^36^, ^37^ ^]^ The impedance spectra can be simulated using loss incorporated equivalent circuit.^[^ ^34^, ^37^ ^]^ The simulation results in Figure 4A,B indicate that the T‐2.5Eu‐2BT exhibits much higher loss than PMN‐PZT‐3BT. Figure S3 (Supporting Information) simulates the effect of different types and different magnitudes of losses on the impedance phase angle. It was found that all losses, especially electromechanical loss tan *γ* = 1/*Q*_m_, have significant impact on the phase angle *θ*_Z,max_. To further confirm this analysis, the effect of Eu^3+^ dopant content on the dielectric, piezoelectric properties, and loss factors is investigated. As shown in Figure 4C, with the increase in Eu^3+^ content, the dielectric permittivity *ε*_r_ significantly increases while loss factors (dielectric loss tan *δ*, electromechanical loss 1/*Q*_m_) are also significantly increased. The increase of dielectric permittivity *ε*_r_ benefits the increase of piezoelectric coefficient *d*, while the increase of loss will reduce the impedance phase angle *θ*_*Z*,max_ and the electromechanical coupling *k*. This opposite trends of *ε*_r_ and *k* limit the enhancement of piezoelectric properties *d*.

In order to understand why the losses of PMN‐PT increase significantly with the increase of Eu^3+^ dopant content, it is very important to determine the fundamental contributions to losses. Figure 4D shows the temperature dependent dielectric behavior of the textured ceramics. It can be observed that the T‐2.5Eu‐2BT has lower phase transition, stronger frequency dispersion of dielectric permittivity, and larger dielectric loss than undoped T‐2BT, PMN‐PT,^[^ ^38^ ^]^ and PMN‐PZT (Figure S4, Supporting Information). Similar to the La^3+^ in PZT, Eu^3+^ cations occupy A‐site by replacing the Pb^2+^ in PMN‐PT, resulting in Pb^2+^ vacancies. In this case, Eu^3+^ as the donor dopant imparts a “soft” piezoelectric behavior (enhanced *d*_33_ but increased losses). The large losses include high dielectric loss due to reduced resistivity/increased leakage (Figure S5, Supporting Information) and high mechanical loss (lower mechanical quality factor *Q*_m_ as shown in Figure 4C) due to easier domain wall motion. Since the losses and piezoelectric response in ferroelectric piezoelectrics are closely related to domain wall motions,^[^ ^35^, ^36^ ^]^ Rayleigh analysis was conducted to understand the effect of Eu^3+^ dopant on intrinsic and extrinsic piezoelectric response in textured ceramics. As shown in Figure 4E, the Eu^3+^ dopant not only greatly increases the intrinsic piezoresponse *d*_int_ from 383 pm V^–1^ to 1052 pm V^–1^, but also significantly increases the extrinsic piezoelectric response *αE*_0_ from 136*E*_0_ pm V^–1^ to 693*E*_0_ pm V^–1^. The large extrinsic piezoelectric contributions resulting from the irreversible domain wall motion drive high losses and low electromechanical quality factor *Q*_m_, as observed in Eu^3+^ doped textured ceramics. The losses in ferroelectric piezoelectric materials are strongly related to phase transition and Eu dopant as shown in Figure S7 (Supporting Information). It can be observed that the hysteresis loss reaches maximum near the phase transition temperature, and Eu dopant results in higher hysteresis loss near the room temperature.

### 2.5 Understanding of Loss Mechanism in Ferroelectrics

**Figure 5:**
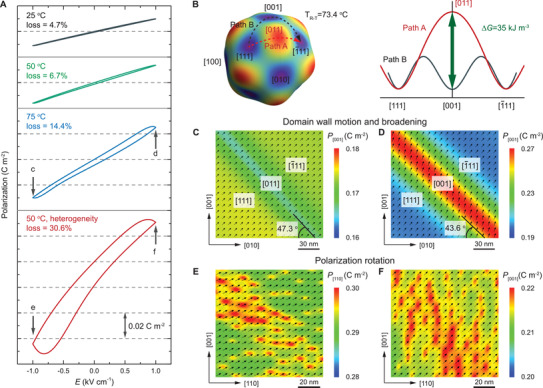
Loss mechanism in PMN‐PT by phase‐field simulation. A) Simulated *P*–*E* hysteresis for the PMN‐PT ferroelectrics with and without heterogenous polar nanoregions at different temperatures, where the poling direction is [001]. B) Landau free energy profiles for two paths of PMN‐PT at rhombohedral‐tetragonal phase transition. C,D) Simulated domain structures corresponding to c and d states of *P*–*E* hysteresis at 75 °C in (A), where domain wall profiles are corresponding to path A and path B in (B), respectively. E,F) Simulated domain structures corresponding to e and f states of *P*–*E* hysteresis at 50 °C with heterogeneity in (A).

The losses in ferroelectric piezoelectric materials are strongly related to ferroelectric domain evolution, which is correlated to phase transition and heterogeneity. To understand the underlying mechanisms of loss in ferroelectrics, we perform phase‐field simulations to study the polarization behaviors induced by electric field at the domain level. We first investigate the temperature dependence of losses in undoped ferroelectrics and then examine the heterogeneity effect in doped ferroelectrics. **Figure** **5**A shows the temperature‐dependent polarization hysteresis induced by electric field *E* that is applied along [001] direction. As the temperature increases from 25 °C (room temperature) to 75 °C (around the R‐T phase transition temperature), the loss is increased from 4.7% to 14.4% for undoped ferroelectrics. Generally, polarization rotation and domain wall motion are the two major mechanisms responsible for the loss in ferroelectrics, which also depends on frequency.^[^ ^39^, ^40^ ^]^ Our simulations reveal that the loss is mainly contributed by domain wall motion while the polarization rotation contribution is almost negligible (at relatively low frequency), because of the faster response of polarization rotation stimulated by electric field. The position of a domain wall depends on the polarization direction which can be changed easily by electric field. Once the polarization is rotated, the domain wall energy is increased accordingly and therefore, the domain wall will adjust its position to reduce the energy. Upon the removal of electric field, the polarization can return to its original state “immediately,” but the domain wall needs more time to come back, especially for long domain walls. In fact, unless the domain wall returns to the original position, the polarization will never come back to its exact initial state, which is the origin of hysteresis. Domain wall vibrations have lower influence on determining the hysteresis as these motions are reversed immediately upon removal of the field. Figure 5C,D illustrate the domain wall motion under electric field. The domain wall angle is changed from 47.3° to 43.6° as the electric field increased from −1 to 1 kV cm^–1^. As the temperature increases, polarization rotation becomes easier and hence the domain wall motion is enhanced, which eventually results in the highest loss at the phase transition temperature. Besides domain wall motion, the domain wall broadening also plays a role. At room temperature, the 71° domain wall is narrow, while at the phase transition temperature, the domain wall becomes broader, based on our simulation results. Figure 5C,D show the domain wall broadening induced by electric field, associated with the change of domain wall type. Since a broader domain wall can overcome pinning sites more easily, it can further enhance the domain wall motion process and thus increase the loss. Figure 5B illustrates the origin of domain wall broadening at the phase transition temperature *T*_R‐T_ = 73.4 °C according to Landau theory. At the phase transition, the tetragonal (T) phase possesses the same energy as the rhombohedral (R) phase, so that it will experience a lower energy if the polarization follows Path B instead of Path A. To examine the heterogeneity effect in doped ferroelectrics, the hysteresis at 50 °C (near the R‐T phase transition temperature *T*_R‐T_ = 48.9 °C for PMN‐PT with 5% heterogeneity) is considered, which exhibits a loss as large as 30.6%. In contrast to the undoped ferroelectrics, the polarization rotation process is significant and can't be neglected due to the heterogeneity that significantly reduces the anisotropy. As shown in Figure 5E,F, the inclusion of heterogeneities makes the polarizations rotate more easily, especially those within heterogeneities, which results in the large hysteresis and thus the large loss.

## 3 Conclusion

In summary, based on phenomenological models and phase‐field simulations, a synergistic design comprising of local structural heterogeneity and grain orientation/texturing engineering is proposed to enhance piezoelectricity via flattening the energy landscape of polarization. This design is exemplified in Eu^3+^‐doped Pb(Mg_1/3_Nb_2/3_)O_3_‐PbTiO_3_ (PMN‐PT) textured ceramics that shows the highest small‐signal piezoelectric coefficient *d*_33_ of up to 1950 pC N^–1^ and large‐signal piezoelectric coefficient *d*_33_* of ≈2100 pm V^–1^. Underlying mechanisms of loss at the domain level is further discussed, which provides a clearer and more comprehensive picture to design high‐performance piezoelectric materials. Ultrahigh performance piezoelectric ceramics, as reported here, will enhance the performance of existing devices and open the possibility of developing new actuation and transduction applications. Since the processing of all ceramic materials reported here follows the traditional multi‐layer manufacturing process, it is expected that these materials will be equally cost‐effective and scalable. Thus, it is expected that these materials will have tremendous impact on advancing the application domain.

## 4 Experimental Section

### Synthesis of [001]_PC_‐Textured and Eu^3+^‐Doped PMN‐PT Ceramics

A series of Pb_(1−1.5_ *_x_*_)_Eu*_x_* [(Mg_1/3_Nb_2/3_)_0.72_Ti_0.28_]O_3_ matrix powders with *x* = 0–0.035, were synthesized by conventional solid state reaction method. Mixture of PbO (99.9%, Sigma Aldrich, USA), Eu_2_O_3_(99.9%, Sigma Aldrich, USA), MgNb_2_O_6_ (99.9%, Alfa Aesar, USA), and TiO_2_ (99.9%, Sigma Aldrich, USA) was ball‐milled in ethanol for 24 h using ZrO_2_ milling media (Tosoh USA). After drying process, the ball‐milled mixture was dried and calcined at 750 °C for 2 h. Calcined powder was ball‐milled again with 1.5 wt% excess PbO for 24 h. The templates for texturing Eu^3+^ doped PMN‐PT ceramics are plate‐like [001]_PC_ BaTiO_3_ (BT) microcrystals. To synthesize the [001]_PC_ BT templates, three steps were involved. First, Bi_4_Ti_3_O_12_ platelets were synthesized by reacting Bi_2_O_3_ with TiO_2_ powders in NaCl and KCl molten salts at 1050 °C for 1 h. Next, BaBi_4_Ti_4_O_15_ platelets were synthesized by reacting Bi_4_Ti_3_O_12_ with TiO_2_ and BaCO_3_ in BaCl_2_/KCl molten salts at 1050 °C for 3 h. Finally, [001]_PC_ BT platelets were obtained by topochemical reaction between BaBi_4_Ti_4_O_15_ and BaCO_3_ in NaCl/KCl molten salt at 950 °C for 3 h. Bi^3+^ in BaBi_4_Ti_4_O_15_ was substituted by the Ba^2+^ from BaCO_3_, yielding BaTiO_3_ template and Bi_2_O_3_ by‐product. The Bi_2_O_3_ by‐product was removed by diluted nitric acid. It should be mentioned here that although a small amount of Bi remains in the lattice of BaTiO_3_ template (Bi/Ba is less than 1/20 as shown in Figure S8, Supporting Information), the synthesized BaTiO_3_ template has perovskite phase structure (Figure S9, Supporting Information), high aspect ratio morphology (Figure 2C), and good thermal stability (Figure 2C,D) in PMN‐PT based matrix, which can be used for fabricating textured ceramics in this study. To fabricate textured ceramics, Eu^3+^ doped PMN‐PT matrix powders with organic binder (Ferro 73225, Vista, CA) and toluene/ethanol solvents were mixed by ball‐milling to prepare ceramics slurry. Next, various contents of BaTiO_3_ templates were added into the slurries under magnetic stirring and the slurries were subsequently casted at the rate of 40 cm min^–1^ by using doctor blade with height of 200 µm. The dried green tapes were cut, stacked, and laminated at 75 °C under 20 MPa pressure for 15 min. The green samples were heated to 400 °C with a heating rate of 0.3 °C min^–1^ and held for 2 h to remove organic solvent and binder, and then isostatically pressed under 133 MPa for 1 min. Samples were subsequently sintered at 1175 °C for 10 h in flowing O_2_ (0.2 L min^–1^).

### Microstructure Characterization

The crystallographic phase and microstructures of ceramics were characterized using X‐ray diffraction (XRD, PANalytical Empyrean) and scanning electron microscopy (FESEM, Apreo) in combination with electron backscatter diffraction (EBSD). The degree of pseudo‐cubic [001] texture was determined from the XRD pattern in 2*θ* range of 20–60° by Lotgering factor method.^[^ ^41^ ^]^ The local microstructure of Eu^3+^ doped PMN‐PT and interface between textured grain and BT template were observed by FEI Titan^3^ G2 double aberration‐corrected microscope at 300 kV. The STEM images were collected by using a high‐angle annular dark field (HAADF) detector which had a collection angle of 52–253 mrad. EDS elemental maps of the sample were collected by using a SuperX EDS system under STEM mode. The TEM sample was prepared by focused ion beam (FIB, FEI Helios 660) lift‐out technique. The atomic positions in the HRTEM images were determined by using two‐dimensional Gaussian fitting by Atomap software.^[^ ^42^ ^]^

### Dielectric and Piezoelectric Property Measurement

The dielectric properties of unpoled and poled samples were measured as a function of temperature by using a multi‐frequency LCR meter (Keysight E4980AL). The piezoelectric properties of samples were obtained by resonance and anti‐resonance technique using impedance/gain phase analyzer (Keysight E4990A, USA) and *d*_33_‐meter (YE 2730 A, APC Products, Inc., USA). The polarization versus electric field hysteresis loops and strain versus electric field curves were measured using ferroelectric Tester (Precision Premier II, Radiant Technologies, Inc., USA).

### Phase‐Field Modeling and Simulations

Phase‐field model of polycrystal ferroelectrics is adopted to perform the simulation study of piezoelectric response of PMN‐PT ceramics by dopant engineering and texture engineering. In particular, in the phase‐field simulations of textured ferroelectric polycrystals, the model of templated grain growth was employed to simulate the grain growth process associated with template seeds, and also the model of template‐matrix composites was employed to simulate the piezoelectric properties of textured doped PNM‐PT ceramics. The details on the model of templated grain growth can be found in the Supporting Information. In the model of template‐matrix composites, the polycrystal grain structure was characterized by the grain rotation matrix field **R**(**r**) while the ferroelectric state was described by the polarization vector field **P**(**r**). The total free energy under externally applied electric field **E** ^ex^ is

(1) $$\begin{array}{ccc} F & = & \int d^{3}r\left[f\left(R_{ij}P_{j}\right)+\frac{\beta }{2}\frac{\partial P_{i}}{\partial r_{j}}\frac{\partial P_{i}}{\partial r_{j}}-E_{k}^{\mathrm{ex}}P_{k}\right]\\ & & +\;\frac{1}{2}\int \frac{d^{3}k}{{\left(2\pi \right)}^{3}}\left[\frac{n_{i}n_{j}}{\varepsilon_{0}}{\tilde{P}}_{i}{{\tilde{P}}_{j}}^{*}+K_{ijkl}{\tilde{\varepsilon }}_{ij}^{0}\tilde{\varepsilon }{{}_{kl}^{0}}^{*}\right] \end{array}$$

where

(2) $$\begin{array}{ccc} f\left(P\right) & = & \alpha_{1}\left(P_{1}^{2}+P_{2}^{2}+P_{3}^{2}\right)+\alpha_{11}\left(P_{1}^{4}+P_{2}^{4}+P_{3}^{4}\right)\\ & & +\;\alpha_{12}\left(P_{1}^{2}P_{2}^{2}+P_{2}^{2}P_{3}^{2}+P_{3}^{2}P_{1}^{2}\right)+\alpha_{111}\left(P_{1}^{6}+P_{2}^{6}+P_{3}^{6}\right)\\ & & +\;\alpha_{112}\left[P_{1}^{4}\left(P_{2}^{2}+P_{3}^{2}\right)+P_{2}^{4}\left(P_{3}^{2}+P_{1}^{2}\right)+P_{3}^{4}\left(P_{1}^{2}+P_{2}^{2}\right)\right]\\ & & +\;\alpha_{123}P_{1}^{2}P_{2}^{2}P_{3}^{2} \end{array}$$

is the Landau–Ginzburg–Devonshire (LGD) free energy of the ferroelectric single crystal, and the coefficients of *α*_*i*_, *α*_*ij*,_ and *α*_*ijk*_ are functions of **r**. The operation *R_ij_P_j_* in *f*(*R_ij_P_j_*) in Equation (1) transforms **P**(**r**) from the global system to the local system in each grain. The gradient term in Equation (1) characterizes energy contributions from polarization gradient in domain wall regions. The **k**‐space integral terms characterize the long‐range electrostatic energy of polarization distribution **P**(**r**) and electrostatic energy of misfit strain distribution ***ε*** ^0^(**r**). The evolution of the polarization **P**(**r**,*t*) is characterized by the time‐dependent Ginzburg–Landau equation

(3) $$\frac{\partial P\left(r,t\right)}{\partial t}=-L\frac{\delta F}{\delta P\left(r,t\right)}$$

where *L* is kinetic coefficient. More details on the phase‐field models of templated grain growth and template‐matrix composites can be found in refs. [43, 44].

In this study, the matrix is piezoelectric PMN‐30PT which has a rhombohedral phase at room temperature, the templates are made of single‐crystalline BaTiO_3_, and the local polar nanoregions within the matrix favor the orthorhombic phase. Therefore, the following LGD coefficients are adopted in the phase‐field simulations: *α*_1_ = 0.745(*T*+160.45)×10^5^ m/F, *α*_11_ = −0.50×10^8^ m^5^/C^2^F, *α*_12_ = −0.5125×10^8^ m^5^/C^2^F, *α*_111_ = 0.5567×10^9^ m^9^/C^4^F, *α*_112_ = 1.333×10^9^ m^9^/C^4^F, *α*_123_ = 0.24×10^9^ m^9^/C^4^F for the PMN‐30PT matrix;^[^ ^45^ ^]^ *α*_1_ = 3.34(*T*‐381)×10^5^ m/F, *α*_11_ = 4.69(*T*‐393)×10^6^–2.02×10^8^ m^5^/C^2^F, *α*_12_ = 3.23×10^8^ m^5^/C^2^F, *α*_111_ = −5.52(*T*‐393)×10^7^ + 2.76×10^9^ m^9^/C^4^F, *α*_112_ = 4.47×10^9^ m^9^/C^4^F, *α*_123_ = 4.91×10^9^ m^9^/C^4^F for the BaTiO_3_ templates;^[^ ^46^ ^]^ *α*_1_ = 2.2(*T*‐560)×10^5^ m/F, *α*_11_ = −2.67×10^8^ m^5^/C^2^F, *α*_12_ = 2.67×10^8^ m^5^/C^2^F, *α*_111_ = 3.33×10^9^ m^9^/C^4^F, *α*_112_ = 3.33×10^8^ m^9^/C^4^F, *α*_123_ = 2×10^10^ m^9^/C^4^F for the local polar nanoregions.^[^ ^12^ ^]^ *T* is the temperature in kelvin. The electrostrictive coefficients are *Q*_11_ = 0.055 m^4^C^–2^, *Q*_12_ = −0.023 m^4^C^–2^, and *Q*_44_ = 0.0315 m^4^C^–2^.

## Conflict of Interest

The authors declare no conflict of interest.

## Supporting information

Supporting Information

## Data Availability

The data that support the findings of this study are available in the supplementary material of this article.
