# Supplementary material for: Ultrahigh Piezoelectric Performance through Synergistic Compositional and Microstructural Engineering
Source: Adv Sci (Weinh). 2022 Mar 16;9(14):2105715. doi: 10.1002/advs.202105715 (PMC9109061; doi:10.1002/advs.202105715)
Supplement: Supplementary file 1 — Supporting Information [file ADVS-9-2105715-s001.pdf]

ADVANCED SCIENCE
Open Access

# Supporting Information

for *Adv. Sci.*, DOI 10.1002/advs.202105715

Ultrahigh Piezoelectric Performance through Synergistic Compositional and Microstructural Engineering

*Yongke Yan*, Liwei D. Geng*, Li-Feng Zhu, Haoyang Leng, Xiaotian Li, Hairui Liu, Dabin Lin, Ke Wang, Yu U. Wang* and Shashank Priya**

# Supporting Information

Ultrahigh piezoelectric performance through synergistic compositional and microstructural engineering

*Yongke Yan[1]*, Liwei D. Geng[2]*, Li-Feng Zhu[1], Haoyang Leng[1], Xiaotian Li[1], Hairui Liu[1], Dabin Lin[3], Ke Wang[3], Yu U. Wang[2]*, Shashank Priya[1]**

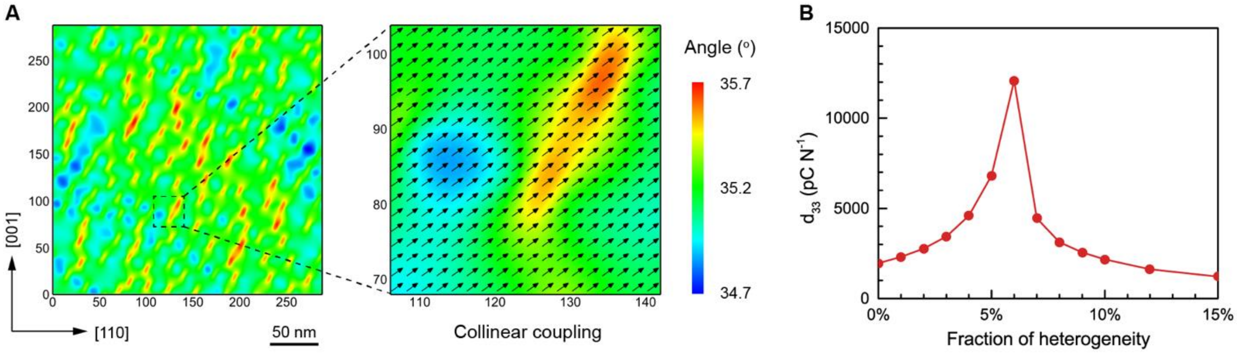


**Figure S1.** Volume fraction effect of local structural heterogeneity on piezoelectricity by phase-field simulation. A) Simulated domain structure of PMN-PT with 5% local structural heterogeneity at room temperature. B) Simulated piezoelectric coefficient $d_{33}$ with different volume fractions of local structural heterogeneity.

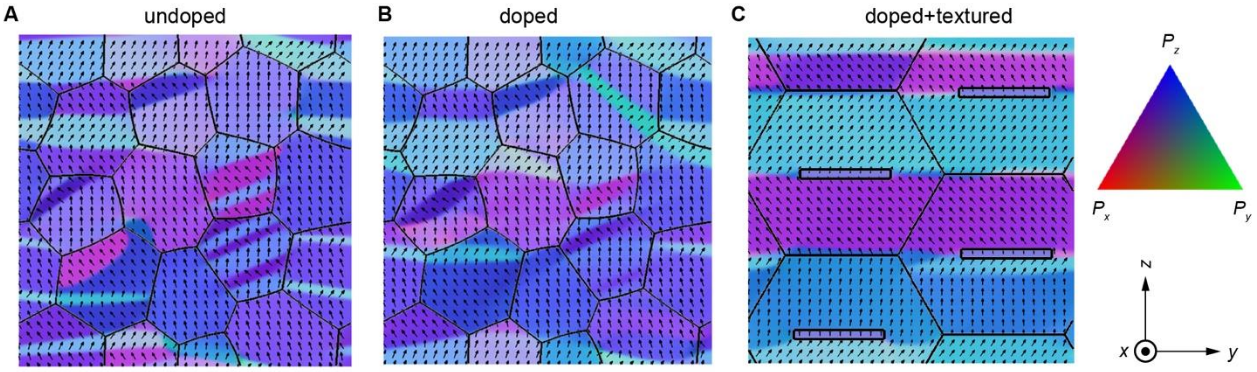


**Figure S2.** Poled ferroelectric domain structures by phase-field simulation. A) Simulated domain structure of undoped PMN-PT ceramic. B) Simulated domain structure of doped PMN-PT ceramic. C) Simulated domain structure of doped and $[001]_{PC}$-textured PMN-PT ceramic with templates made of $BaTiO_3$.

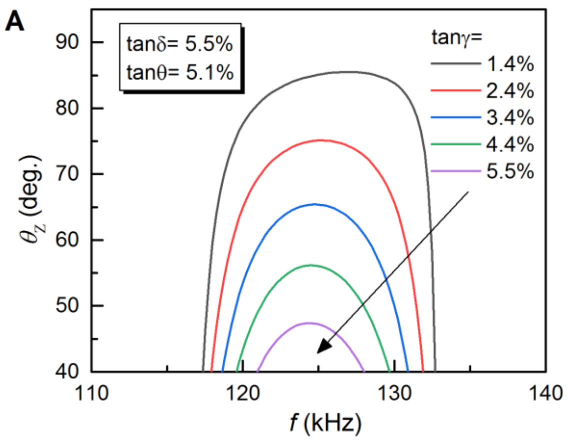


**Figure S3.** Simulated impedance phase angle $\theta_Z$ spectra with different loss factors.

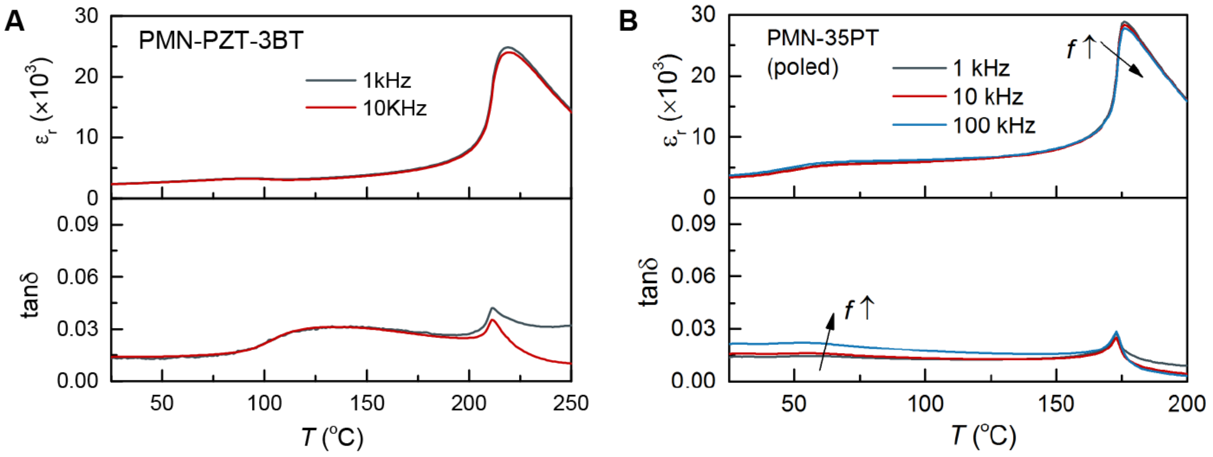


**Figure S4.** Temperature dependent dielectric constant $\varepsilon_r$ and dielectric loss tanδ. (A) <001> textured 40PMN-25PZ-35PT ceramics with 3 vol% $BaTiO_3$ template, abbreviated as PMN-PZT-3BT. (B) PMN-35PT random ceramics.

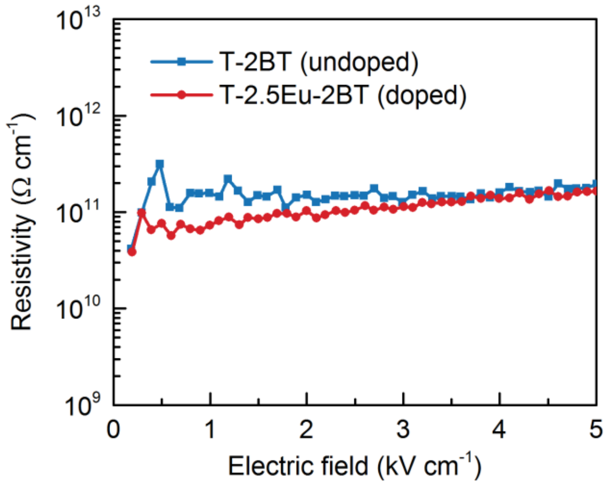


**Figure S5.** Resistivity of T-2BT (undoped) and T-2.5Eu-2BT (doped) textured ceramics.

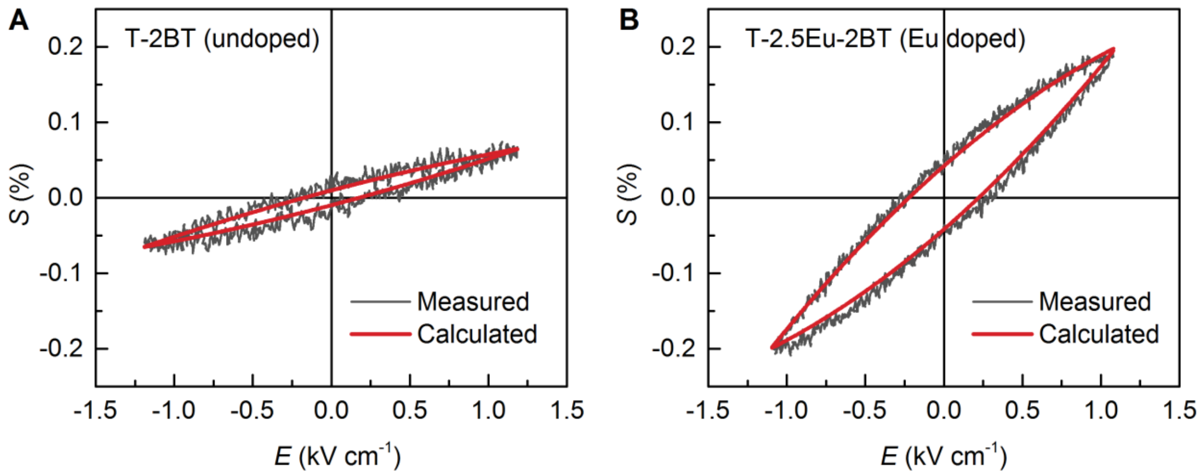


**Figure S6.** Comparison between the measured and calculated strain-vs-electric field hysteresis loops. A) T-2BT (undoped) ceramics and B) T-2.5Eu-2BT (doped) ceramics.

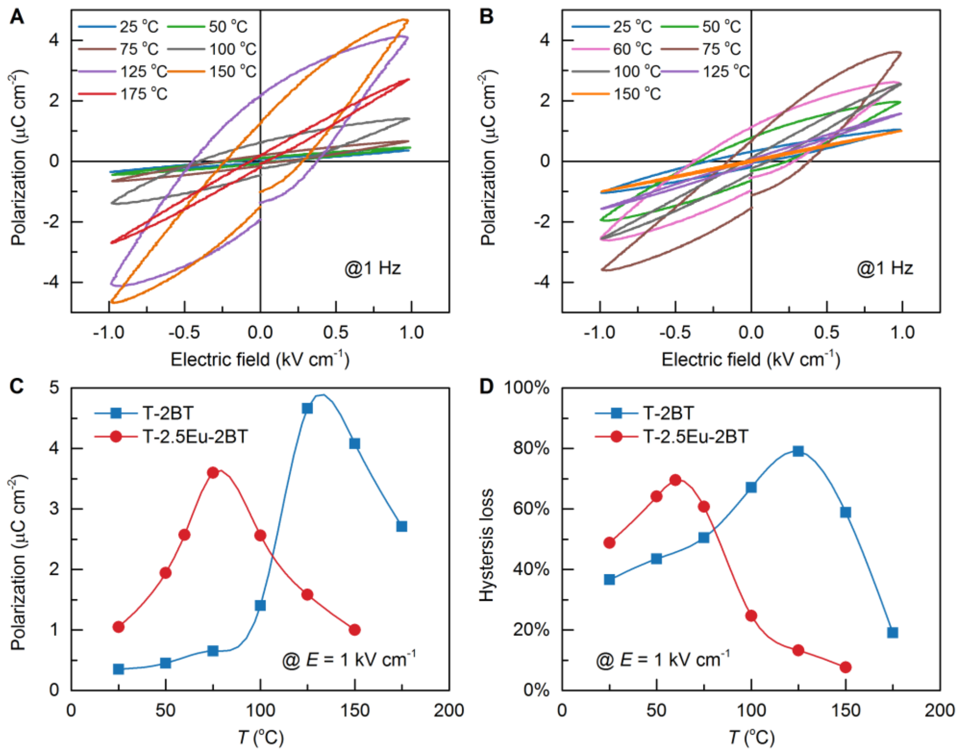


**Figure S7.** Polarization-electric field (*P*-*E*) hysteresis. (A) *P*-*E* hysteresis loops for the T-2BT (undoped) ceramics at different temperatures. (B) *P*-*E* hysteresis loops for the T-2.5Eu-2BT (doped) ceramics at different temperatures. (C) Polarization at 1 kV $cm^{-1}$ at different temperature. (D) Hysteresis loss at 1 kV $cm^{-1}$ at different temperature.

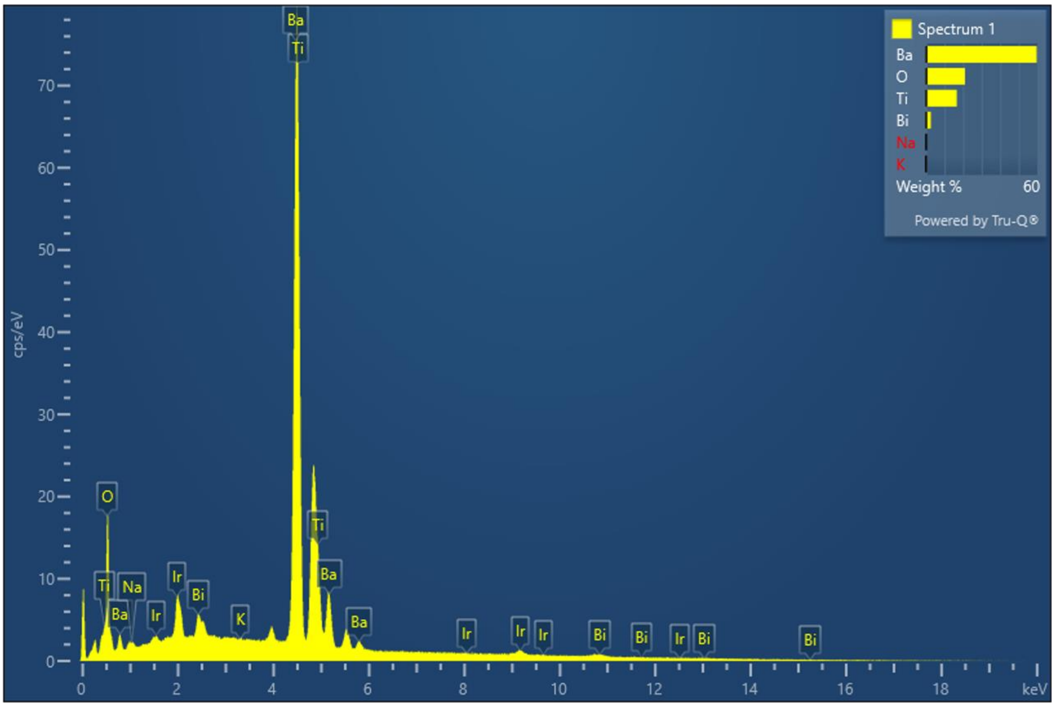


| Element | Line Type | Wt% | Atomic % |
|---|---|---|---|
| O | K series | 21.04 | 62.26 |
| Na | K series | 0.07 | 0.14 |
| K | K series | 0.03 | 0.03 |
| Ti | K series | 16.61 | 16.41 |
| Ba | L series | 59.63 | 20.55 |
| Bi | M series | 2.62 | 0.59 |
| Total: | | 100.00 | 100.00 |

**Figure S8.** EDS analysis of $BaTiO_3$ template.

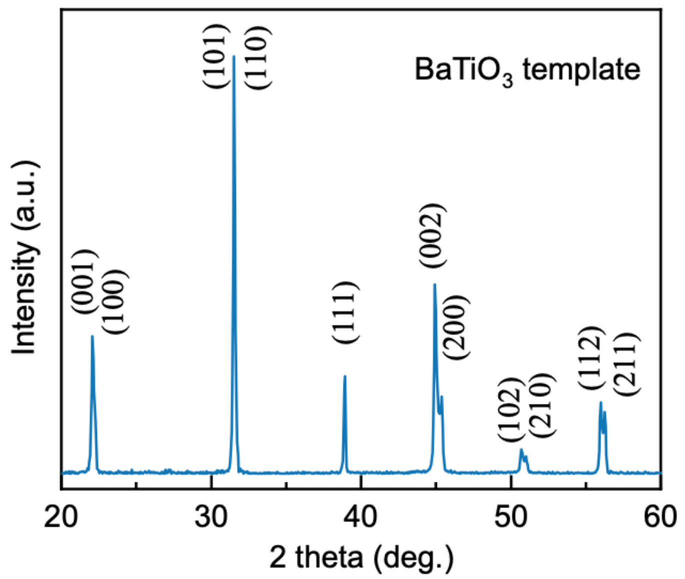


**Figure S9.** XRD pattern of $BaTiO_3$ template.

**Supplementary Text**

**Phase-field modeling of templated grain growth**

In the model of templated grain growth, the grain microstructure is described by a set of long-range order parameter field variables $\{\eta_\alpha(\mathbf{r})\}$. The total free energy is

$$F = \int d^3r \left[ f\left(\{\eta_\alpha\}\right) + \sum_\alpha \frac{1}{2}\beta \left|\nabla \eta_\alpha\right|^2 \right], \tag{1}$$

where $f\left(\{\eta_\alpha\}\right)$ is the Landau-type polynomial potential function that is formulated as

$$f\left(\{\eta_\alpha\}\right) = A\left[\sum_\alpha \left(3\eta_\alpha^4 - 4\eta_\alpha^3\right) + \sum_{\alpha \neq \alpha'} 6\eta_\alpha^2 \eta_{\alpha'}^2\right]. \tag{2}$$

The gradient term characterizes energy contribution from grain boundaries. The grain growth process is governed by the time-dependent Ginzburg-Landau equation

$$\frac{\partial \eta_\alpha(\mathbf{r},t)}{\partial t} = -L\frac{\delta F}{\delta \eta_\alpha(\mathbf{r},t)} + \xi_\alpha(\mathbf{r},t), \tag{3}$$

where $L$ is the kinetic coefficient and $\xi_\alpha(\mathbf{r},t)$ is the Langevin noise term that characterizes the thermal fluctuation effect. In the simulation process, the long-range order parameter fields of template seeds are not evolved, but these templates can induce heterogeneous nucleation and heteroepitaxial growth of neighboring grains onto them.

**Loss calculation from measured impedance spectra**

In the extensional vibration of a rectangular bar, the admittance can be approximated as (*34*)

$$Y = \frac{j\omega lw}{t}\left(\varepsilon_{33}^T - \frac{d_{31}^2}{s_{11}^E}\right) + \frac{j2wd_{31}^2}{\left(\rho s_{11}^E\right)^{1/2} s_{11}^E t}\tan\frac{\omega l\left(\rho s_{11}^E\right)^{1/2}}{2}, \tag{4}$$

when the length ($l$), width ($w$), and thickness ($t$) of the bar satisfy $l \gg w$, $l \gg t$, and $w > 3t$. Here, $j = \sqrt{-1}$ is the imaginary unit, $\omega = 2\pi f$ is the angular frequency, $\rho$, $s_{11}^E$, $d_{31}$, and $\varepsilon_{33}^T$ are the density, the elastic compliance constant, the piezoelectric constant, and the permittivity component in the length mode of the piezoelectric bar. The impedance is $Z = 1/Y$, and its phase angle is $\theta_Z = \arctan(\mathrm{Im}\,Z\,/\,\mathrm{Re}\,Z)$, where "Re" and "Im" represent the real and imaginary parts, respectively.

**Rayleigh analysis of textured ceramics**

Rayleigh analysis was performed on undoped and doped textured ceramics. The Rayleigh law is expressed using the following equations:

$$S(E) = ((d_{init} + \alpha E_0)\,E \pm \alpha(E_0^2 - E^2)/2, \tag{5}$$

$$d(E_0) = (d_{init} + \alpha E_0)\,pm/V, \tag{6}$$

where $S(E)$ is the field induced strain, $d_{init}$ is the intrinsic piezoelectric response, $E_0$ is the maximum applied field, $E$ is the applied field, $\alpha$ is the irreversible Rayleigh parameter resulting from the irreversible motion of internal interfaces, and $\alpha E_0$ represents the extrinsic contribution to the total piezoelectric response. Figure S6 shows the measured and calculated strain-vs-electric field hysteresis loops using Equation (5) for T-2BT (undoped) ceramics and T-2.5Eu-2BT (doped) ceramics.

**References for Figure 3E**

(1) Random ceramics

**Commercial PZT4, PZT5A,PZT5H,PZT8:** Exelis Inc. Electro-Ceramic Products and Material Specification (2011).

**Sm-PMN-29PT**: F. Li, D. B. Lin, Z. B. Chen, Z. X. Cheng, J. L. Wang, C. C. Li, Z. Xu, Q. W. Huang, X. Z. Liao, L. Q. Chen, T. R. Shrout, S. J. Zhang, *Nature Materials* **2018**, 17, 349.

(2) Textured ceramics

**PIN-PMN-PT**: Y. F. Chang, B. Watson, M. Fanton, R. J. Meyer, G. L. Messing, *Appl. Phys. Lett.* **2017**, 111, 232901.

**PMN-PT**: Y. K. Yan, Y. U. Wang, S. Priya, *Appl. Phys. Lett.* **2012**, 100, 192905.

**PYN-PMN-PT**: Y. F. Chang, J. Wu, Z. Liu, E. W. Sun, L. J. Liu, Q. W. Kou, F. Li, B. Yang, W. W. Cao, *ACS Appl. Mater. Interfaces* **2020**, 12, 38415.

**PMN-PZT**: Y. K. Yan, K. H. Cho, D. Maurya, A. Kumar, S. Kalinin, A. Khachaturyan, S. Priya, *Appl. Phys. Lett.* **2013**, 102, 042903.

**PNN-PZT**: L. Bian, X. Qi, K. Li, Y. Yu, L. Liu, Y. F. Chang, W. W. Cao, S. X. Dong, *Adv. Funct. Mater*. **2020**, 30, 2001846.
